# Quantifying and reducing the cost of tagging: combining computational fluid dynamics and diving experiments to reduce impact from animal-borne tags

**DOI:** 10.1098/rspb.2024.1441

**Published:** 2024-11-06

**Authors:** J. Chris McKnight, Chris Pass, Dave Thompson, Steve Balfour, Sophie M. J. M. Brasseur, Clare Embling, Gordon Hastie, Ryan Milne, Adam Kyte, Simon E. W. Moss, Richard Pemberton, Debbie J. F. Russell

**Affiliations:** ^1^ Sea Mammal Research Unit (SMRU), Scottish Oceans Institute, University of St Andrews, St Andrews, Fife KY16 8LB, UK; ^2^ Plymouth University, Drake Circus, Plymouth PL4 8AA, UK; ^3^ Sea Mammal Research Unit Instrumentation Group, Scottish Oceans Institute, University of St Andrews, St Andrews, Fife KY16 8LB, UK; ^4^ Department of Ecology, Wageningen Marine Research, Den Helder PO Box 57, The Netherlands

**Keywords:** tag effects, CFD, telemetry, biologging, diving animals, 3 Rs

## Abstract

Animal-borne instruments are essential research tools for ecologists and physiologists. An increasing number of studies have shown impacts of carrying a tag on behaviour and energetics, which can have implications for animal welfare and data validity. Such impacts are a result of the additional mass and/or drag loads, with the latter requiring empirical measurements or computational fluid dynamics (CFD) to estimate. To quantify and effectively minimize tag impacts from drag, a novel combined empirical and CFD approach is required. Here, we demonstrate such an approach using captive phocid seals and the widely used Sea Mammal Research Unit (SMRU) Instrumentation Group GPS/GSM tag. We (i) show a significant change in the behaviour of grey seals when carrying a tag (gen 1; associated with 16.4% additional drag); (ii) redesigned the tag (gen 2) resulting in a lower additional drag of 8.6%; (iii) show significant differences in behaviour when carrying a gen 2 compared to gen 1 tag, demonstrating that the redesign successfully reduced impact; and (iv) observed changes in the swim speed of seals that were consistent with predictions from CFD estimates of drag. The gen 2 instrument is now commercially available. This non-trivial case study should pave the way for similar studies in other taxa and species.

## 1. Introduction

Deploying tags on animals is an integral part of modern ecological research and conservation. Notwithstanding the overwhelming benefit that devices provide to pure and applied research, it has been shown that carrying external tags can adversely affect tagged individuals. Two key factors underpinning the adverse impact of carrying external devices are (i) tag mass and (ii) the additional drag forces that tags can cause. For all species, except perhaps large whales, mass has been the focus of concern, and thus research. However, for aquatic and flying species, additional drag from external tags is also a key concern, and for marine mammals it is the main concern [1]. Thus, while existing knowledge for the impact of instrument mass on study animals has led to the creation, and utilization, of ‘rules of thumb’ (e.g. 3% and 5% rules as applied to mass), there is comparatively very little information on how the effects of drag should be considered/minimized in tagging practices.

Additional drag from external tags has been shown to affect the behaviour and energetics of instrumented animals. These impacts can have implications for the costs of foraging [2], which, in turn, may ultimately affect the long-term survival of tagged individuals [3]. Yet, compared to impacts of mass, impacts of drag have only been investigated in a limited number of species such as Stellar sea lion (*Eumetopias jubatus*) [1], northern fur seal (*Callorhinus ursinus*) [4], bottlenose dolphin (*Tursiops truncatus*) [5], king penguin (*Aptenodytes patagonicus*) [6] and great cormorant (*Phalacrocorax carbo*) [7]. The mass of an external tag is a simple metric, whereas quantifying additional drag is a complicated, dynamic metric affected by multiple factors. Nevertheless, it is incumbent on researchers to reduce the impact that biologging devices have; first from an ethical standpoint to improve animal welfare and second from a scientific standpoint, to reduce potential biases in data (electronic supplementary material, table S5). Therefore, researchers need to increase efforts to estimate, and ultimately minimize, the impact of tag drag.

Both pressure and viscous drag must be considered when examining drag tag and its minimization. The total drag force acting on an animal moving in water or in flight is the sum of these two dynamic components. Pressure drag (sometimes termed ‘form’ or ‘profile’ drag) results from the pressure differential between the front and rear of a body as it moves through the fluid. Viscous (or skin friction) drag arises owing to friction between the fluid and the body surface and is caused by the fluid viscosity [8]. Generally, most of the additional drag associated with a tag is owing to pressure drag. Indeed, pressure drag is likely to dominate in any case where the tag is relatively ‘bluff’—and thus boundary layer separation from the surface of the body forms an appreciable low-pressure wake. Viscous drag is strongly influenced by the boundary layer flow regime (i.e. whether it is laminar, partly turbulent or fully turbulent) [9]. As most aquatic and flying species have evolved bodies that closely approximate the ideal axisymmetric, streamlined form, laminar to turbulent transition within the boundary layer is often assumed to occur at the widest point of the body [9]. The change in viscous drag induced by a tag is dependent mainly upon where on the body of an animal a tag is placed.

Computational fluid dynamics (CFD) modelling can be used to estimate the baseline drag of an animal travelling through a medium, along with the additional pressure and viscous drag from a tag [6], and ultimately to inform on the best tag placement and design that will minimize drag [7]. In general, tags should be placed on the posterior of an animal, away from the transition zone; this will minimize any increase in viscous drag induced by a tag by allowing the transition to occur closer to its natural location [10,11]. However, to collect locational data for marine mammals, tags generally need to be placed close to the breathing apparatus, which is in front of the transition zone, resulting in a relatively large increase in viscous drag [12] because the tag induces boundary layer transition. Although it is still important to estimate viscous drag in these cases, efforts to minimize drag have to focus on the minimization of pressure drag. Studies using CFD modelling to estimate drag and ultimately improve the hydrodynamic performance of tags to reduce pressure drag are vitally important [12–14]. However, there is an absence of knowledge of how tag drag translates to observable effects at the animal level. Yet, it is against metrics such as behaviour and energetics that tag impacts and design improvements must be measured.

**Figure 1.**
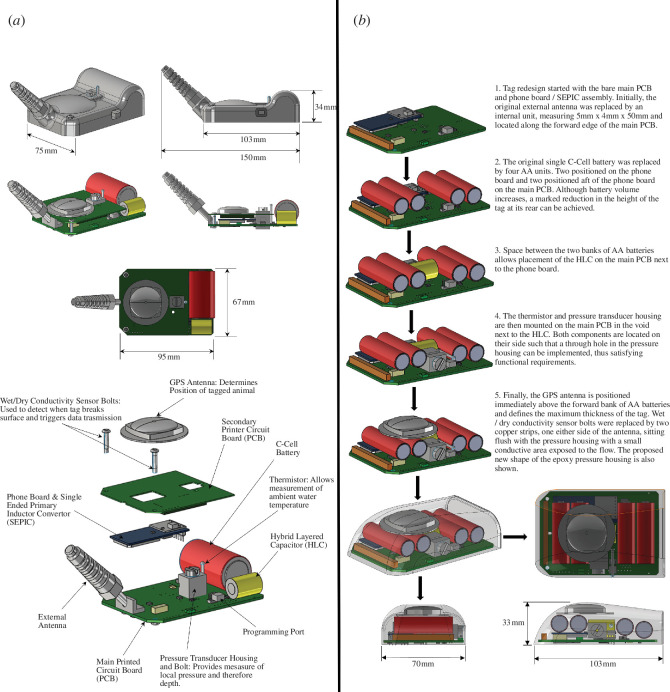
(*a*) Generation 1 (gen 1) GPS phone tag dimensions, components and layout. (*b*) The step by step detail of component placement/packaging within the proposed new gen 2 tag. The proposed new epoxy housing is also shown and illustrates the removal of the external antenna.

To address the above gaps in knowledge, we present a non-trivial case study focusing on a phocid seal (grey seal; *Halichoerus grypus*) and a GPS/GSM tag made by the Sea Mammal Research Unit (SMRU) Instrumentation Group. The first generation (hereafter gen 1) of this instrument (figure 1*a*) has been available since 2006, selling more than 1400 units by the time of publication. In this study, we aim to (i) describe a process to quantify the behavioural and energetic impacts of carrying a tag associated with a known drag (from CFD modelling [6]), (ii) use CFD to redesign the tags (gen 2) to minimize drag, (iii) quantify the behavioural and energetic differences between carrying a gen 1 and gen 2 tag, and (iv) compare CFD-predicted and empirically measured differences in swim speeds associated with carrying a gen 1 tag, compared with a gen 2 tag and no tag.

## 2. Methods

### (a) Captive animal work (aims 1, 3 and 4)

All experiments were conducted with wild-caught grey seals (cohort 1: six caught in 2013 and cohort 2: five caught in 2015). Cohorts 1 and 2 were used to address aims 1 and 3, respectively. Data from both cohorts were used to address aim 4. All animals were temporarily housed at the SMRU Pool Facility (see [15]).

Epoxy replicas of the gen 1 tags, with no internal components, were produced. Tags were attached to the animal using a polyurethane baseplate to allow repeated attachment and detachment of the replica tag. Baseplates matched the footprint of the tag and were 6 mm thick, resulting in baseplate dimensions of 103 mm × 75 mm × 6 mm for gen 1 tags and 103 mm × 60 mm × 6 mm for gen 2 tags. To account for this extra height, the replica tags were 6 mm shallower than the functioning tags. In control (‘untagged’) treatments, seals dived without the replica instrument but with the baseplate.

Animals were anaesthetized to allow attachment of a baseplate that remained on the animals throughout the experiments. Animals were pre-treated with midazolam (Hypnovel, Roche Products, UK; 5 mg ml^−1^ solution at a dose rate of 0.03 ml per kg intramuscular (IM)) and subsequently anaesthetized with either Zoletil® (Zoletil 100, Virbac, Australia; 100 mg ml^–1^ solution at a dose rate of 0.1 ml per 20 kg intravenous (IV)) or ketamine (Ketaset, Zoetis, UK; 100 mg ml^−1^ solution at a dose rate of 0.1 ml per 10 kg IV) [16]. The baseplate was bonded to the fur on the back of the neck, at the base of the skull, using superglue (Loctite® 4861, Henkel, UK). The placement of the baseplate was chosen to replicate the usual placement of gen 1 tags on free-ranging seals [17]. Replica tags were attached with two cable ties that passed through channels in the baseplate and in the body of the tag.

#### (i) Simulated foraging set-up

Seals were trained to dive either 58 m or 110 m (set-up varied between years owing to logistical constraints) from a breathing chamber (connected to an open-flow respirometry system) in one corner of the experimental pool, to reach a feeding device that acted as an artificial ‘prey patch’ in the opposite corner (see [18] for details). The feeding device comprised an aluminium-framed conveyor belt that ran from the surface to a feeding window *ca* 2 m below the surface. The belt had an elasticated covering that held fish in place so that as the belt turned, fish were made available to the seal at the feeding window. Belt speed and distance between fish on the belt remained constant throughout the trials to ensure a constant rate of fish delivery until the daily ration was consumed.

An important aspect of the simulated foraging system was that during trials, seals were free to determine how long they remained at the feeder, how fast they transited to and from the feeder and how long they remained at the surface breathing. Seals were typically exposed to each treatment (no tag, gen 1 and gen 2) for a seven-day block, before being handled to change treatment. Each trial consisted of multiple dives on a single day. A trial ended when the animal had consumed a pre-determined daily maintenance ration of fish (*Sprattus sprattus*) from the conveyor belt in the feeding station, so the duration of trials was determined by the time taken to consume the daily food ration at the feeder. The mass of fish that each seal consumed was kept constant across all experimental treatments and was dependent on an individual’s dietary requirements.

#### (ii) Measuring behaviour

During each dive, the seal swam from the breathing chamber to the feeding station (referred to as descent time (s)), spent time at the feeder (bottom time (s))—when they were usually stationary while removing fish from the conveyor belt—swam from the feeding station back to the breathing chamber (ascent time (s)) and finally spent time in the breathing chamber before the next dive (post-dive surface interval; PDSI (s)). Underwater cameras (Sony IR 37CSHR-IR 25 m) at the feeding station and breathing chamber were connected to a digital video recorder (DVR) system and allowed accurate measurement of descent time, bottom time, ascent time and PDSI. The dive during which the daily ration was finished was removed from subsequent analyses.

#### (iii) Measuring oxygen consumption rate

The breathing chamber formed part of an open-flow respirometry set-up that allowed estimation of the total oxygen consumption for each dive cycle. The structure of the respirometry set-up, oxygen measurement protocols and the one-step nitrogen dilution calibration method used in this study have been described in detail by Sparling *et al*. [18]. Oxygen consumption (*V*O_2_) (i.e. the total amount of oxygen consumed over a dive cycle (l)) was calculated using the following equation [19]:

,$$VO_{2}=(0.2094VN_{2}/0.8)(\Delta C/\Delta C_{*})$$

where Δ*C* and ΔC^*^ refer to the deflection of the analyser during measurement and calibration, respectively, and *V*N_2_ is the volume of nitrogen used in the calibration. Oxygen consumption (*V*O_2_) was estimated for each dive cycle by dividing the *V*O_2_ by the total dive cycle time. Mass-specific oxygen consumption (l kg^−1^) was calculated by dividing *V*O_2_ by animal mass.

#### (iv) Analysis

Analyses were conducted to quantify the difference in behaviour and energetics of diving seals when carrying a gen 1 tag versus no tag (aim 1) and a gen 1 versus a gen 2 tag (aim 3). Owing to logistical constraints, the starting treatment (gen 1 versus no tag or gen 1 versus gen 2) could not be randomized across individuals. Preliminary data exploration indicated an initial impact of experience (i.e. days since the start of the first trial) on behavioural metrics, which varied by individual. Data exploration and initial analyses also indicated that there was substantial variation in responses across dives within a trial, with a potential temporal trend in some instances. This prohibited the fitting of robust models to the raw data. Thus, a conservative approach was taken to address these issues. First, to avoid confounding between treatment and experience, only the last four treatment blocks were retained. This resulted in the removal of the first one or first two blocks, and experience days were included in the model (see electronic supplementary material, table S6). Second, only the median response in each trial was retained for each behavioural metric per trial.

For each cohort, five maximal models were considered, one for each of the metrics described above (descent time, bottom time, ascent time, PDSI and oxygen consumption). Each response metric was modelled as a function of treatment and behavioural, physiological and extrinsic covariates (electronic supplementary material, table S6). The framework used for analysis was generalized additive mixed-effects models (GAMM) constructed within the ‘mgcv’ package in R (v. 1.8-35) [20]. Additive models were used to allow complex, nonlinear relationships between covariates and response metrics. Mixed-effects models were required to account for the non-independence of data within individual seals (by having an individual as a random intercept term) and to investigate individual variation in the impact of treatment (through an interaction between treatment and individual). These random effects were not subject to model selection. Each maximal model also included experience as a global smooth function with an individual-level smooth function (using a factor-smoother interaction; model 2 in [21]), allowing a shared trend with an individual-level trend with similar smoothness to the shared trends. This restriction on smoothness ensured that the experience 'smooth' did not account for variation caused by treatment. Maximal models for cohort 1 included an interaction between treatment and distance to account for the two distances used (58 m and 110 m). Maximal models for each metric are given in electronic supplementary material, table S1.

For each metric, the final model (electronic supplementary material, table S1) was chosen by a backward stepwise model selection process minimizing the Akaike’s information criterion (AIC [22]), with parameters excluded if their inclusion did not improve the model by 2 or more ΔAIC. The global smooth of experience was only subject to selection if model selection resulted in the removal of the individual-level smooth. Treatment was not subject to selection but rather the significance of the term in the final model was assessed to address the relevant aims. Random effects (individual), and the interaction with treatment, were not subject to selection. Residual plots were checked for evidence of violation of model assumptions.

### (b) Development of gen 2 tag (aim 2)

#### (i) Gen 1 redesign

To fulfil aim 2, we used the findings of Kyte *et al*. [12] along with computed aided design (CAD) to design a new tag (gen 2) within the functional design constraints of the hardware.

The gen 1 tag (figure 1*a*) was rectangular in platform and the main printed circuit board (PCB) determined its footprint (103 mm × 75 mm, excluding the external antenna). The highest point of the tag body was governed by the diameter of the C-cell battery, located close to the rear of the tag. CFD modelling by Kyte *et al*. [12] estimated that this gen 1 tag generated *ca* 16.4% additional drag on a model swimming seal moving at a constant speed of 1.3 m s^−1^, 10% and 90% of which were from viscous and pressure drag, respectively. Although some of this pressure drag was attributable to the external antenna that had an 18 mm base radius, 18 mm tip radius and was 70 mm in length, the bulk was generated by the low-pressure wake formed as flow separates from the curved surface of the C-cell battery casing forming the caudal surface of the instrument [6].

The aim here was to redesign the tag to reduce pressure drag while retaining all hardware and functionality. To avoid increasing the contact surface on tagged animals, it was determined that the footprint of gen 2 should not exceed that of gen 1. A key design constraint was that the main PCB had to constitute the base of the tag with the phone board/single ended primary inductor convertor (SEPIC) assembly remaining in the same position as in gen 1 tags. A full explanation of the component reconfiguration is provided in the electronic supplementary material.

The key aspects of the redesign were: (i) the location of the maximum height of the device was moved forwards to provide a tapered afterbody from the tallest point in order to reduce the height of the tag at its rear, thus reducing wake size; (ii) the external phone antenna was replaced with an internal embedded unit, which removed the disturbance to flow induced by the antennae; and (iii) to minimize disturbance to flow caused by other surface discontinuities.

CAD software (Solidworks 2019, Visiativ Solutions UK) was used to produce dimensionally accurate three-dimensional models of all required internal components, thus allowing multiple component combinations and internal layouts to be assessed. A final proposed gen 2 tag shape was then defined based on the most suitable, space-efficient component layout. Figure 1*b* details the process taken to develop the revised layout and shows the final gen 2 tag shape defined by the epoxy pressure housing.

#### (ii) Gen 2 tag shell and dimensions (in comparison with gen 1)

The gen 2 tag fore-body tapers up in size to a maximum height of 33 mm, defined by the dorsal position of the GPS antenna, before tapering down in thickness towards the rear of the tag at an angle of 11°. The location of maximum thickness was moved forwards, to *ca* 42 mm downstream of the leading edge. Consequently, maximum thickness was reduced by 3% and height at the tag's trailing edge was reduced by 31% (to 23 mm). Additionally, a reduction in the thickness of the epoxy coating on the tag's sides means that the width was reduced by *ca* 7%. Placing the batteries and GPS antenna centrally along the width of the PCB meant that large radii fillets could be incorporated to shape the tag around the internal components, thus reducing the bluffness of the tag.

#### (iii) Computational fluid dynamics (CFD) methods

Our CFD methods followed those in Kyte *et al*. [12], which considered the drag associated with no tag and gen 1 tags. However, our study considered the drag associated with gen 2 only. The CFD modelling is outlined here, but it is described in greater detail in the electronic supplementary material, where additional mesh dependency checks are also detailed.

An accurate three-dimensional model of a free swimming, adult female harp seal (*Pagophilus groenlandicus*) in the glide phase travelling at a constant speed of 1.3 m s^−1^ was used. This three-dimensional seal model was developed following the work of Kyte *et al*. [12]. To our knowledge, no such models of grey seals exist. However, the fineness ratio (ratio of length to maximum width) is similar between the grey and harp seal, as are their average transit speeds (1.3 m s^−1^).

The animal model measured 1.85 m in length; it is 0.44 m in diameter at its widest point and the front of the tag base is located 0.25 m behind the animal’s nose. The animal was assumed to be hydro-dynamically smooth and rigid, with small geometric features such as the vibrissae removed. Fore flippers were merged with the body of the animal and the closed paired hind-flippers were merged into a single body. The tag was aligned with the direction of the oncoming flow. Details on assumed environmental conditions are provided in electronic supplementary material, table S2. Simulation boundary conditions are provided in electronic supplementary material, table S3.

#### (iv) Comparison of CFD-predicted changes and empirically measured changes

In order to assess the ability of CFD model outputs to estimate biologically relevant tag effects, a simple energetics model was constructed and used to determine (i) the increase in output power required when wearing both gen 1 and gen 2 tags, compared with no tag, in order to maintain the specified swim speed of 1.3 m s^−1^ and (ii) the reduction in swim speed that would be required under gen 1 tag and gen 2 tag conditions to maintain the untagged power output.

For an animal swimming at a constant speed in rectilinear motion, a simplified estimate of the mean output power (*P*) required to sustain a given average velocity (*U*) is given by the drag-based model shown in equation (2.1), in which *F*_*x*_ equals the mean total drag force predicted by simulation. The time (*t*) taken to swim a distance (*D*) is given by equation (2.2).


(2.1) $$P=\;F_{x}\cdot U$$

(2.2) $$t=\frac{D}{U}.$$

The increase in output power (watts) required to maintain the set swim speed is determined by applying equation (2.1) to simulation results for each different treatment (no tag, gen 1 and gen 2).

In order to estimate the reduction in swim speed necessary to maintain the untagged power output under gen 1 and gen 2 tag treatments, it is assumed that output power is proportional to *U*^3^ as per equation (2.3), in which λ is a constant of proportionality relating swim speed and output power. Since *F*_*x*_ and therefore *P* are different for untagged, gen 1 and gen 2 tag conditions, a specific λ value can be determined for each. Consequently, the new, *reduced* swim speed at which output power equals that of the 'no tag' condition can be estimated for gen 1 and gen 2 tag conditions in turn by rearranging equation (2.3) and using the untagged power output and relevant λ value as inputs:


(2.3) $$P=\;\lambda U^{3}.$$

## 3. Results

### (a) Aim 1: quantify the impact of gen 1 tag on behaviour and energetics

The data used in the analyses (post-processing; see §2a(i) were 45 and 39 trials for no tag treatment and tagged treatment, respectively (summarized by the six individuals in electronic supplementary material, table S4).

The presence of the gen 1 tag resulted in significantly slower descent (*p *< 0.01) and ascent speeds (*p *< 0.01), and significantly longer bottom times (*p *< 0.01; compared to diving with no tag); the direction of response was consistent across individuals. There was no significant difference in PDSI or oxygen consumption with tag presence.

#### (i) Descent speed

**Figure 2.**
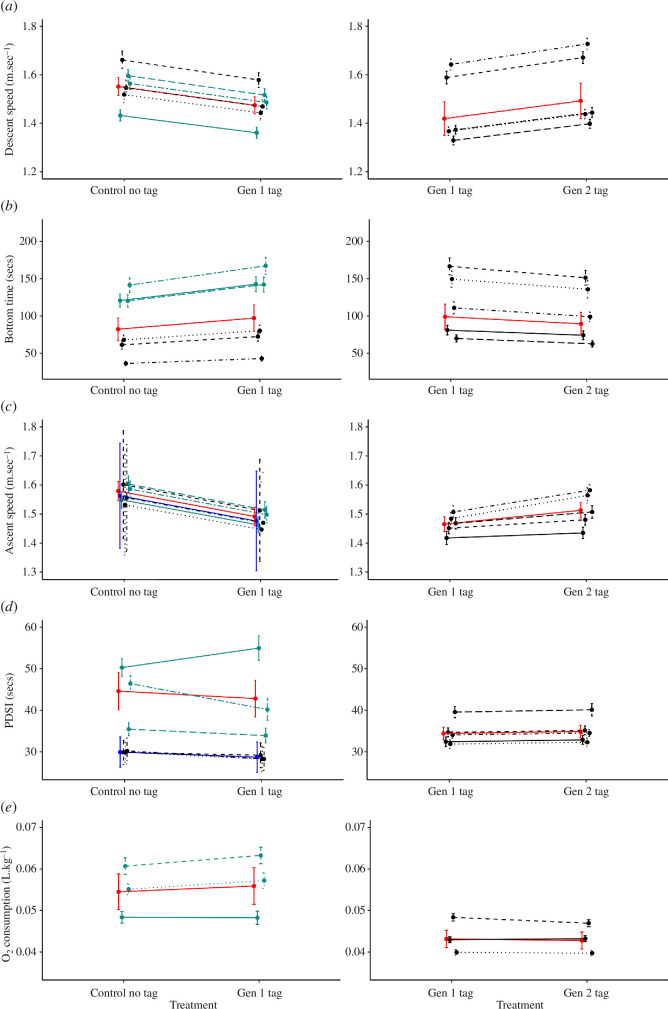
Comparison of the effects of diving without a tag and with the gen 1 tag (left panels), and diving with the gen 1 tag and gen 2 tag (right panels) on (*a*) descent speed, (*b*) bottom time, (*c*) ascent speed, (*d*) post-dive surface interval and (*e*) mass-specific oxygen consumption. Left and right panels do not include the same individual seals. Black lines represent responses of individual seals transiting 58 m and green lines 110 m transits. Red lines represent average responses. The presence of a blue line in the left-hand panel represents average responses on 58 m transits and red lines 110 m transits, where transit distance was retained by model selection.

Descent speed was *ca* 5.4% slower when seals were tagged (gen 1); 1.55 m s^−1^ ± 0.3 (standard error) and 1.47 m s^−1^ ± 0.3 without and with a gen 1 tag, respectively (figure 2*a*). The global smooth for experience day was retained, but not the individual-level smooth. Distance was not retained.

#### (ii) Ascent speed

Ascent speed was *ca* 5.8% slower when seals were tagged (gen 1): 1.56 m s^−1^ ± 0.18 and 1.47 m s^−1^ ± 0.17 for a 58 m transit without and with a gen 1 tag, respectively (figure 2*c*). The global and individual-level smooth for experience day was retained, as well as the interaction between distance and bottom time (and thus their component parts). The interaction between distance and treatment was not retained (electronic supplementary material, table S1).

#### (iii) Bottom time

Mean bottom time was *ca* 18% longer when seals were tagged (gen 1); 82.34 s ± 15.1 and 97.28 s ± 17.7 without and with a gen 1 tag, respectively (figure 2*b*). The global and individual-level smooth for experience day, distance and descent speed were not retained (electronic supplementary material, table S1).

#### (iv) PDSI

Treatment was not significant (*p *> 0.1; figure 2*d*). The global and individual-level smooth for experience day, distance and bottom time were retained. Transit duration (ascent and descent duration combined) and the interaction between distance and treatment distance were not retained (electronic supplementary material, table S1).

#### (v) Oxygen consumption

Although there was no significant difference in oxygen consumption by treatment, the direction of mean response was consistent across individuals with lower oxygen consumption when tagged (figure 2*e*). PDSI was retained, while transit duration, bottom time and the global and individual-level smooth for experience day were not retained (electronic supplementary material, table S1).

### (b) Aim 2: development of gen 2 tag

Table 1 presents mean total drag predictions (both as absolute forces and as drag coefficients (*C*_d_) for an animal fitted with the gen 2 tag. These were compared with drag predictions for both an untagged animal and an animal fitted with the gen 1 tag (from Kyte *et al*. [12]). Both fully turbulent and transitional results are given in table 1*a*,*b*, respectively. In addition, the *total* predicted drag forces are broken down to show the components attributable to the animal body and to the tag.

**Table 1.** CFD-predicted drag forces showing: (*a*) fully turbulent model results and (*b*) transitional model results.

(*a*) fully turbulent (shear stress transport (SST) model only)			
	untagged animal	animal + current tag	animal + generation 2 tag
mean total drag force (N)	9.720	10.810	9.849
mean tag drag force (N)	—	1.080	0.144
mean animal body drag force (N)	9.720	9.740	9.705
drag coefficient (*C*_d_FT_)	0.0062	0.0069	0.0063
percentage increase in mean total drag force versus untagged animal	—	11.21%	1.33%
percentage reduction in total reduction drag force versus current tag	—	—	8.89%

(*b*) transitional (shear stress transport transition model (SST-y))			
	untagged animal	animal + current tag	animal + generation 2 tag
mean total drag force (N)	8.19	9.52	8.908
mean tag drag force (N)	—	1.11	2.44
mean animal body drag force(N)	8.19	8.41	8.664
drag coefficient (*C*_d_TR_)	0.0052	0.0061	0.0057
percentage increase in mean total drag force versus untagged animal	—	16.24%	8.77%
percentage reduction in total reduction drag force versus current tag	—	—	6.43%

**Figure 3.**
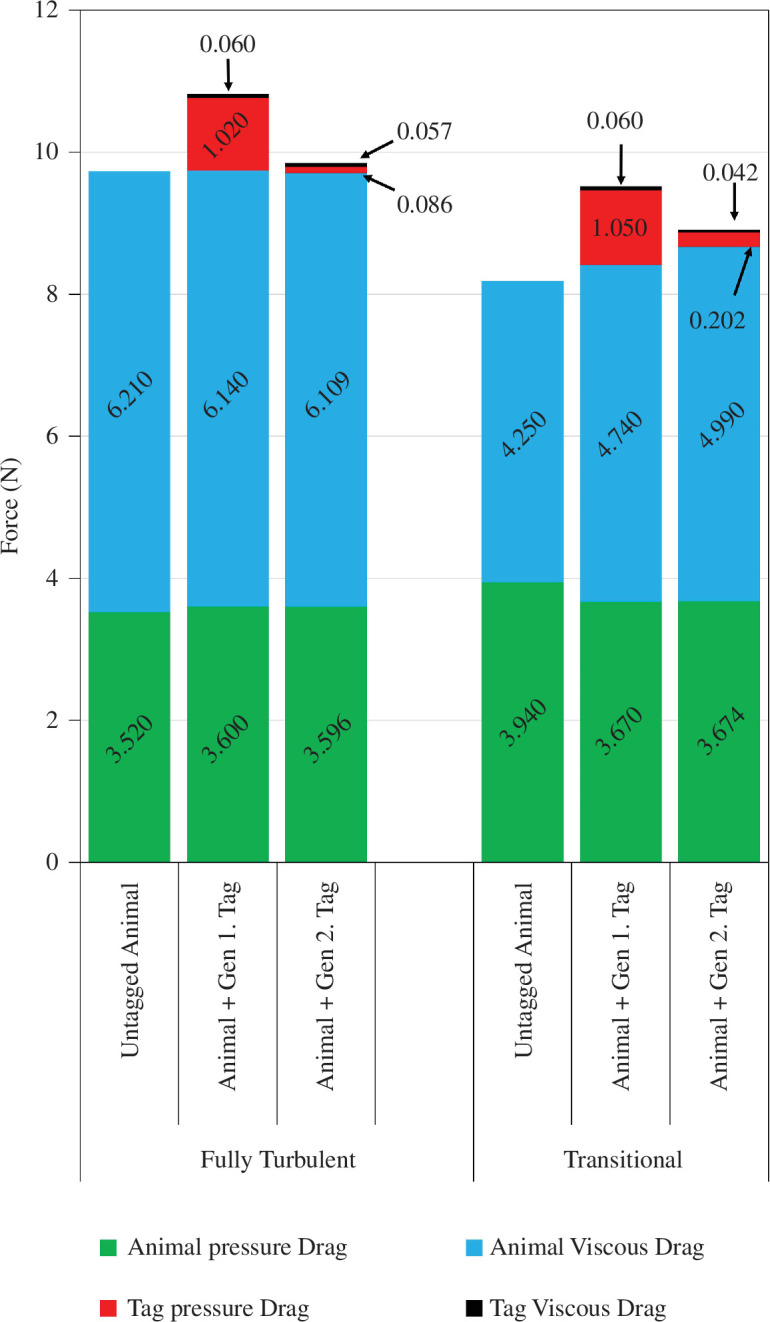
Comparison of pressure and viscous drag components for cases in which the animal is untagged, fitted with the genn 1 tag and finally, fitted with the gen 2 tag. In all cases, the simulated swim speed is 1.3 m s^−1^.

Figure 3 breaks these mean forces down further, into viscous and pressure drag components for the animal body and, where fitted, for both gen 1 and gen 2 tags.

The gen 2 tag shape generated markedly less hydrodynamic drag than the gen 1 tag, with reductions of around 86% and 77% predicted by the fully turbulent model and transitional model, respectively. Fully turbulent model results predicted that the gen 2 tag yields a 91% reduction in tag pressure drag while viscous drag remains approximately equal to that of the gen 1 tag. The transitional model predicted 80% and 30% reductions in tag pressure and viscous drag, respectively. The gen 2 tag yields *total* (i.e. seal + tag) drag reductions of 8.89% and 6.43% compared to a seal fitted with the gen 1 tag, under fully turbulent and transitional conditions, respectively.

### (c) Aim 3: comparison of the impact of generation 1 and generation 2 phone tags on behaviour and energetics

The data used in the analyses (post-processing; see §2) were 55 trials each for the gen 1 and gen 2 treatments, respectively (summarized by the five individuals in electronic supplementary material, table S4).

Compared to a gen 1 tag, the presence of the gen 2 tag resulted in significantly quicker descent (*p *< 0.01) and ascent speeds (*p *< 0.05) and significantly shorter bottom times (*p *< 0.01). However, there was no significant difference in PDSI or oxygen consumption.

#### (i) Descent speed

Mean ascent speed was *ca* 4.9% faster when swimming with the gen 2 compared with the gen 1 tag: 1.42 m s^−1^ ± 0.70 and 1.49 m s^−1^ ± 0.70 with the gen 1 and gen 2 tag, respectively (figure 2*a*). The direction of response of descent speed was consistent across individuals.

The global and individual-level smooth for experience day were retained (electronic supplementary material, table S1).

#### (ii) Ascent speed

Mean ascent speed was *ca* 2.7% faster when swimming with the gen 2 compared to gen 1 tag: 1.47 m s^−1^ ± 0.02 and 1.51 m s^−1^ ± 0.01 with a gen 1 and gen 2 tag, respectively (figure 2*c*). The direction of response of ascent speed was consistent across individuals. The global and individual-level smooth for experience day and descent speed were retained during model selection. Bottom time was removed (electronic supplementary material, table S1).

#### (iii) Bottom time

Mean bottom time was estimated to be *ca* 9.9% shorter when diving with the gen 2 compared to gen 1 tag: 99.03 s ± 17.0 and 89.54 ± 15.4 with a gen 1 and gen 2 tag, respectively (figure 2*b*). The direction of response of bottom time was consistent across individuals. The global and individual-level smooth for experience day, treatment and descent rate were retained during model selection (electronic supplementary material, table S1).

#### (iv) PDSI

PDSI was not significantly different (*p *> 0.1) when diving with the gen 2 and gen 1 tags. The direction of the response of PDSI (increase with gen 2 compared to gen 1 tag) was consistent across individuals (figure 2*d*). Experience day at an individual-level smooth, treatment and dive duration were retained during model selection (electronic supplementary material, table S1).

#### (v) Oxygen consumption

Mass-specific oxygen consumption was not significantly different (*p *> 0.1) when diving with the gen 2 and gen 1 tags. The direction of response of oxygen consumption varied across individuals. PDSI, experience day at an individual-level smooth and bottom time were retained during model selection. Transit duration was not retained (electronic supplementary material, table S1).

### (d) Aim 4: comparison of CFD-predicted changes and empirically measured changes

Applying equations 2.1, 2.2 and 2.3 to the fully turbulent simulation results shown in table 1*a* indicates that an output power of 12.64 W is required to sustain a swim speed of 1.3 m s^−1^ under untagged conditions, while 14.05 W and 12.80 W are required under gen 1 and gen 2 tag conditions, respectively. The time taken to cover the 58 m distance employed during experiments would be 44.6 s. Applying the same calculations to the transitional simulation results given in table 1*b* yields required respective power outputs of 10.65, 12.38 and 11.58 W. As the speed is fixed, the transit time would necessarily be the same.

Applying equation 2.3, CFD results predict that, compared to the untagged case, a reduction in swim speed of 3.48–4.89% would be necessary under fully turbulent and transitional conditions, respectively, when wearing the gen 1 tag. Experimentally observed reductions were 5.4% (descent) and 5.8% (ascent, 58 m transit). Diving with the gen 2 tag, CFD results suggest that the swim speed would increase compared to the gen 1 tag condition by 3.15–2.24% under fully turbulent and transitional conditions, respectively, and the experimentally observed increases were 4.9% (descent) and 2.7% (ascent).

## 4. Discussion

In the first of its kind, addressing four aims, this study has shown the impact of carrying a tag (of known drag) on behaviour; how CFD can be used to redesign a functional tag to minimize drag; that such a minimization of drag has implications in terms of behaviour; and finally, the relationship between CFD-predicted changes in movement versus observed changes provide reliable estimates. This study was conducted using a relatively small sample size of grey seal juveniles within a captive facility. Addressing aim 1, we demonstrated that the presence of a tag (gen 1, which was associated with 16.4% additional drag but was only 1% of body weight) resulted in observable changes in behaviour (longer dive times through both longer swim and bottom times). However, there was no significant change in PDSI or oxygen consumption. Addressing aim 2, we used CFD to redesign the tags (gen 2) to minimize drag while maintaining functionality, resulting in an additional drag from the tag of 8.8% (compared to 16.4% for gen 1). Addressing aim 3, we demonstrated that the decreased additional drag associated with a gen 2 tag had observable implications for seal behaviour: when carrying the gen 2 tag, seals had a shorter swim and bottom times, and thus dive durations than when carrying the gen 1 tag. Finally, addressing aim 4, we demonstrated that CFD accurately predicted impact on transit speeds, comparing no tag, gen 1 and gen 2. The gen 2 SMRU GPS/GSM is now commercially available, with *ca* 250 instruments having been deployed on free-ranging phocid seals.

The changes in behaviour associated with carrying a tag are likely a function of the decrease in the speed associated with the minimum cost of transport as a result of the additional drag from the tag [23,24]. Reduced transit speeds under conditions of elevated drag penalties have been reported for other marine vertebrate species, including Adelie penguins (*Pygoscelis adeliae*) [25], northern elephant seals (*Mirounga angustirostris*) [26], bottlenose dolphins [5] and northern fur seals [4]. The increase in bottom time was likely a consequence of the change in transit duration mediated through the change in transit speed. Gallon *et al*. [27] demonstrated that on longer transit durations, grey seals increase residency times (i.e. bottom time) at a prey patch.

Despite these changes in dive duration, there was no detectable impact on PDSI or oxygen consumption. In diving animals, there is an asymptotic relationship between dive duration and PDSI, likely owing to the time to replenish oxygen stores [28]. Increased dive durations resulting from carrying a tag have also been shown to be associated with increased PDSI [6]. The fact that the relationship was not found here, and that the direction of change in PDSI when carrying a tag varied across individuals, suggests that the seals may not have fully replenished oxygen stores during inter-dive intervals. McKnight *et al.* [15] demonstrated that harbour seals (*Phoca vitulina*) do not extend their surface durations to compensate for greater depletion of oxygen stores, and instead develop a growing oxygen debt over a bout of consecutive dives. Seals not fully replenishing oxygen stores between dives may also explain why there was only a marginal effect on oxygen consumption, whereas other studies have reported elevations under higher drag penalties [4]. If the animals do not fully replenish oxygen stores, then the dive-by-dive estimates of oxygen consumption are likely to be underestimates of the total oxygen consumed during each dive. If animals did consume more oxygen when diving with a gen 1 tag, compared to no tag or gen 2, but the animals did not increase PDSI to allow for additional oxygen loading to replenish additional costs (assuming a constant respiration rate and tidal volume) then the amount of oxygen loaded by the seals during the interval (used to measure oxygen consumed in the preceding dive) could appear equal. Therefore, it may be that there is an unaccounted oxygen debt (energetic cost) in the current study that the animal pays off after the completion of a diving bout [15]. Alternatively, at lower arterial *P*O_2_ levels resulting from longer dive durations, the rate of oxygen loading would be higher early in the surface interval. That this did not present as a higher rate of oxygen consumption rate could be because the magnitudes of change in oxygen loading were not of sufficiently high to reach a detection threshold for metabolic rate of oxygen consumption.

Despite the constraints of tag design, it was possible to reduce the additional drag associated with carrying a tag by 52%, which resulted in observable reductions in the impact of carrying a tag on behaviour. Indeed, the tag footprint remained unchanged, and the difference in the mass between the tags was only 2% (gen 1 = 302 g versus gen 2 = 295 g). The key changes here were that the location of the maximum height of the device was moved forwards, the external phone antenna was replaced with an internal embedded one and surface discontinuities were removed to create a smooth, streamlined profile.

CFD-derived estimates of change in swim speed were within a few per cent of those from the model averages from the experimental data. CFD-derived estimates of changes in swim speed did, however, generally underestimate the actual impact of additional tag drag on swim speed. An underestimate of the behavioural impact of additional drag is likely the consequence of CFD underestimating the additional drag from an external tag. CFD modelling is, by definition, conducted under highly idealized conditions, with numerous geometric simplifications required to make a task computationally feasible [12]. A more fundamental issue with CFD, in this study, is the assumption that the animal is rigid, with water flowing only along its longitudinal axis. In reality, this represents only the glide phase of an animal’s swim gait. In phocid seals, for example, which are thunniform swimmers [29], the horizontal beating of the hind-flippers is reciprocated by low-amplitude horizontal oscillations of the head. This has important implications for CFD predictions of flow dynamics around a tagged seal because (i) periodic deformation of the body is likely to markedly alter the hydrodynamic forces acting on the animal, meaning that the tag drag may become a smaller proportion of total drag, and (ii) a gen 1 or gen 2 tag is attached to the neck region on a seal, which means that lateral head movements associated with active swimming will periodically cause the anterior face of the tag to diverge from its longitudinal axis. If the tag is not aligned with the flow along the longitudinal axis, the drag on the tag may increase [30]. Therefore, the drag forces predicted by our CFD model are likely to represent estimates of both the lowest drag experienced by a swimming seal *and* the lowest drag of a tag. Nonetheless, we demonstrate that CFD estimates of changes in swim speed are comparable with experimental observations, giving confidence that (i) results from the CFD modelling effort are reasonable estimates of those realized in animal studies and (ii) CFD can be used to inform tag redesign efforts that provide a realistic approximation of realized amelioration to subject animals.

The changes in behaviour as a result of carrying a tag have implications for data validity and potentially for animal welfare. This study shows both the impacts of carrying tags and that these can be reduced by changes to tag design. For pinnipeds, we speculate that there are likely to be behavioural biases in tagged animals—specifically, slower swim speeds and longer bottom durations, which, when combined, would translate to overall longer dive durations. The impact of this on tagging data is dependent on the specific tag and animals used in the study, as well as the resolution of data produced. For example, given that the relative additional drag from a standard external tag would increase on smaller study animals, the impact of the additional drag tag would be greater for small animals. Ultimately, the impact on research findings will depend on the research question. For example, studies focused on trip metrics, at sea distribution and coarse activity budgets are likely to be more robust than those exploring size-specific differences in dive duration. Critically, combining data on such fine-scale metrics across different tags (e.g. gen 1 versus gen 2) should be done with caution. The impacts of carrying a tag could, in some cases, have welfare implications. Indeed, we speculate that smaller marine mammals or juvenile animals are the most likely to be impacted (in terms of behaviour, energetics and/or fitness) by tag drag, as well as individuals that are already at risk of starvation. However, the risk of impacts for healthy adult animals is likely to be reduced because adults have greater capacity (lower metabolic rates and greater resource storage) to adapt and compensate to changes in their environment, as well as a lower relative additional drag from a given instrument. Indeed, a recent publication assessing the effects of telemetry instruments on adult female grey seals showed no impact on predicted offspring mass or likelihood of breeding in the following year [31]. Similar longitudinal studies on smaller marine mammals and juveniles are outstanding but are essential to better contextualize and assess the risk of telemetry studies on fitness.

Tag drag should be routinely considered in the cost–benefit evaluation conducted for tag studies as part of the 3Rs (replacement, reduction and refinement) [32,33]. To do so effectively, it is critical to be able to quantify and minimize tag drag—then aspects of instrument design could be integrated into the assessment of risk to animal welfare. Wilson & McMahon [33] described the potential impacts of studying animals on three numerical levels: (i) the extent of the detriment; (ii) the number of animals involved; and (iii) the length of time over which the detrimental effects can occur. In support of Wilson and McMahon’s advocation to attempt to quantify these features in study animals and multiply them together to obtain an overall detriment index, we encourage the integration of tag design and the drag associated with a particular instrument into this risk assessment. The current study outlines how, using a combination of models and live animals, this can be conducted to generate values for behavioural and energetic costs; and in part how CFD modelling alone can provide realistic inferences on specific behavioural impacts (swim speed). Moreover, the design principles that we outline here (detailed overview in the electronic supplementary material)—which reduce impact, and therefore improve animal welfare (based on the Bateson cube [32,34])—have broad applicability to existing instrumentation. We hope these principles will be adopted more widely. Incorporating design principles that reduce drag must be part of the broader responsibility of the biologging community and maintaining best animal welfare practices.

In most cases, reduction in tag drag first required quantification of the drag itself and then tag redesign. However, in cases where tag function will not be affected by tag placement, we recommend that external tags are placed as far back on subject animals as possible. Caudal placement of instruments will achieve a reduction of drag without tag redesign efforts. We recommend practical efforts to reduce drag that consider the range of uses of the tag in question. Although we have demonstrated that CFD provides potentially conservative drag estimates, it is the critical step in quantifying and minimizing the impact of tag drag. It can be used to compare the impacts of tag designs on different study species and sizes. Although idealized designs may result in some reduction in drag, this will only exist at a conceptual level (e.g. [14]). Consideration of the tag functionality and design constraints is critical to the generation and uptake of tags that minimize drag and realize the demand for improvement. Efforts here to reduce drag on a commercially available telemetry system have shown that the redesign process can result in realized improvements at the level of animal behaviour—thus we encourage others to attempt similar efforts that will result in more hydrodynamic hardware deployments on free-ranging animals. Our study demonstrates that CFD is a viable tool for ecological applications of drag investigation, offering a methodology that will allow engagement with researchers and tag developers investigating drag-related issues. With this, we strongly encourage that both the research community and tag development groups begin to explore using CFD to attempt to quantify and to understand the impact that current instruments have on drag, and begin more targeted efforts to reduce drag [12–14,35–38].

The limitations associated with CFD highlight the importance of empirical studies to measure changes in behaviour and energetics. Such studies are rare owing to the inherent difficulties of comparing tagged and non-tagged individuals in the wild, and the limited number and capacity of captive facilities. Indeed, our study was based on a limited sample size (six for no tag versus gen 1 and five for gen 1 versus gen 2). Nevertheless, here we found that despite substantial variation in swim speed and bottom durations, the impacts of the tag in terms of magnitude and direction of change were similar across individuals. This suggests that even if researchers can only investigate the impacts of external tags on a small number of individuals, as in the present study, relatively modest changes in additional drag (here 6.4%) can be detected. Indeed, in this case, this was despite logistical constraints resulting in data loss (exclusion of 1 to 2 weeks of data and the use of median values as response metrics). Therefore, we encourage more attempts at experimental assessment of drag-induced impacts, especially since changes in drag will be system-specific, and could feasibly be age- and sex-specific in some species, even if only a small number of study animals are available.

## 5. Conclusion

This study demonstrates that tag-induced drag is a concern and likely induces some behavioural changes to instrumented animals. Despite the complexities in assessing drag, we demonstrate that CFD can be used to inform improved tag design and accurately estimate some of the realized benefits measured in animals diving with the improved tag. This new low drag instrument may, in some cases, have welfare benefits as well, reducing potential behavioural biases caused by an external tag—two key factors that both researchers and instrument developers have a responsibility to improve.

## Data Availability

All data and code are available at [39]. Supplementary material is available online [40].
